# Interstitial Lung Disease Associated with mTOR Inhibitors in Solid Organ Transplant Recipients: Results from a Large Phase III Clinical Trial Program of Everolimus and Review of the Literature

**DOI:** 10.1155/2014/305931

**Published:** 2014-12-18

**Authors:** Patricia Lopez, Sven Kohler, Seema Dimri

**Affiliations:** ^1^Novartis Pharma AG, Postfach, 4002 Basel, Switzerland; ^2^Boehringer Ingelheim GmbH, Binger Straße 173, 55216 Ingelheim, Germany; ^3^Novartis Healthcare Pvt. Ltd., Raheja Mindspace, Hitech City, Madhapur, Hyderabad, Rangareddy 500081, India

## Abstract

Interstitial lung disease (ILD) has been reported with the use of mammalian target of rapamycin inhibitors (mTORi). The clinical and safety databases of three Phase III trials of everolimus in *de novo* kidney (A2309), heart (A2310), and liver (H2304) transplant recipients (TxR) were searched using a standardized MedDRA query (SMQ) search for ILD followed by a case-by-case medical evaluation. A literature search was conducted in MEDLINE and EMBASE. Out of the 1,473 *de novo* TxR receiving everolimus in Phase III trials, everolimus-related ILD was confirmed in six cases (one kidney, four heart, and one liver TxR) representing an incidence of 0.4%. Everolimus was discontinued in three of the four heart TxR, resulting in ILD improvement or resolution. Outcome was fatal in the kidney TxR (in whom everolimus therapy was continued) and in the liver TxR despite everolimus discontinuation. The literature review identified 57 publications on ILD in solid organ TxR receiving everolimus or sirolimus. ILD presented months or years after mTORi initiation and symptoms were nonspecific and insidious. The event was more frequent in patients with a late switch to mTORi. In most cases, ILD was reversed after prompt mTORi discontinuation. ILD induced by mTORi is an uncommon and potentially fatal event warranting early recognition and drug discontinuation.

## 1. Introduction

Interstitial lung disease (ILD) constitutes a heterogeneous group of noninfective lung disorders. Based on etiology, ILD is categorized into nine main groups: idiopathic interstitial pneumonia, connective tissue disease, smoking-related, vasculitis, granulomatous disease, environmental/occupational, drug-induced, inherited, and others [1]. It is the most common form of drug-induced lung toxicity. Various drug classes are known to cause ILD, including chemotherapy agents (e.g., bleomycin, cyclophosphamide, and chlorambucil), cardiovascular drugs (e.g., amiodarone, beta blockers, and statins), anti-inflammatory drugs (e.g., sulfasalazine, gold salts, and methotrexate), antimicrobial agents (e.g., nitrofurantoin and amphotericin), and biological agents (e.g., etanercept and infliximab) [2]. The clinical presentation is similar to that of infectious pneumonia, with dyspnea being the most common symptom. Typical radiological findings include bilateral reticular or reticulonodular opacities. Drug-induced ILD is mainly diagnosed by exclusion of other causes and by a thorough review of drug history, complemented by high-resolution computed tomography (CT), bronchoscopy with bronchoalveolar lavage, and bronchoscopic or surgical lung biopsy. The histopathological findings associated with drug-induced ILD are interstitial pneumonia, hypersensitivity pneumonia, bronchiolitis obliterans organizing pneumonia, and granulomatous pneumonitis. Early diagnosis is crucial since delayed discontinuation of the suspected drug may lead to a fatal outcome.

Diagnosing ILD is particularly challenging in transplant recipients because the nonspecific symptoms of ILD may be attributed to infectious conditions, which are common in this population and because patients are polymedicated. Mammalian target of rapamycin (mTOR) inhibitors are used increasingly in solid organ transplantation due to their synergistic effect with calcineurin inhibitors, which allows for calcineurin inhibitor dose reduction, and their antiproliferative properties [3, 4]. Although occurrence of ILD has been reported in patients receiving the mTOR inhibitors everolimus and sirolimus [5, 6], the condition is uncommon and thus is difficult to evaluate as an endpoint in randomized controlled trials. Published reports of mTOR inhibitor-induced ILD largely comprise single cases or retrospective analyses of patient cohorts which supply limited information regarding diagnostic criteria and use varying terminology to describe the condition. A larger data set based on consistent criteria would provide useful information regarding the incidence, management, and outcome of ILD in mTOR inhibitor-treated patients.

We performed a systematic search of clinical and safety data from three large Phase III clinical trials of everolimus in* de novo* kidney, heart, and liver transplant recipients. The studies, although conducted in different types of solid organ transplantation, had many similarities with regard to study design, observation period, and inclusion of a control group. Furthermore, each trial applied stringent quality criteria to obtain regulatory approval and registration of everolimus. In addition, we performed a literature review of ILD cases associated with everolimus or sirolimus to assess prevailing clinical practice for the diagnosis and management of ILD in solid organ transplant recipients.

## 2. Methods

### 2.1. ILD in Clinical Trials of Everolimus

We evaluated ILD cases from adverse events reported during three prospective, randomized, 24-month trials of everolimus designed to evaluate the efficacy and safety of everolimus in* de novo* kidney (A2309), heart (A2310), and liver transplant (H2304) patients. The study designs and results have been reported previously [7–9]. Of the 2,273 patients randomized in the A2309 (*n* = 833), A2310 (*n* = 721), and H2304 (*n* = 719) studies, 1,473 patients received everolimus either in combination with reduced calcineurin inhibitor therapy or as monotherapy.

In the A2309 study, patients were randomized to receive everolimus 0.75 mg b.i.d (target trough concentration [C_0_] 3–8 ng/mL) or 1.5 mg b.i.d. (C_0_ 6–12 ng/mL) with reduced-dose cyclosporine, or mycophenolic acid (MPA, 1.44 g/day) in combination with standard-dose cyclosporine. All patients received induction with basiliximab. The first dose of study drug was administered within 24 hours after transplantation. In the A2310 study, patients were randomized to receive everolimus 0.75 mg b.i.d (C_0_ 3–8 ng/mL) or 1.5 mg b.i.d (C_0_ 6–12 ng/mL) with reduced-dose cyclosporine or mycophenolate mofetil (MMF) 3 g (1.5 mg b.i.d.) with standard-dose cyclosporine within 72 hours of transplantation. Centers chose from three induction strategies: (1) basiliximab 20 mg administered on days 0 and 4 after transplant; (2) rabbit antithymocyte globulin administered as per local practice, starting on the day of transplantation; or (3) no induction. In the H2304 study, liver transplant recipients were randomized, after a 30- (±5-) day run-in period with tacrolimus (±MMF), to everolimus (C_0_ 3–8 ng/mL) with reduced tacrolimus (C_0_ 3–5 ng/mL) or everolimus (C_0_ 6–10 ng/mL) with tacrolimus withdrawal at month 4 or standard exposure tacrolimus (C_0_ 6–10 ng/mL). In all three studies, corticosteroids were initiated at the time of transplantation and administered as per local practice, with optional corticosteroid tapering after six months in the H2304 study.

To identify cases of drug-induced ILD, the clinical and safety study databases were first searched for adverse event terms included in the standardized MedDRA query (SMQ) search for ILD (Table 1). Each identified case was reviewed by the study clinician and the medical safety expert for medical history, clinical presentation, concurrent conditions, concomitant medication, diagnostic test results, treatment, and outcome of the event. Predefined criteria for exclusion of drug-induced ILD were as follows: diagnosis of pulmonary infection, response/resolution of event with antibiotics, mild events with spontaneous resolution, diagnosis of other pulmonary condition, off-study medication at the time of onset of respiratory syndrome and insufficient information to establish the etiology. Only cases qualified as drug-induced ILD are presented and discussed in detail.

### 2.2. Literature Review

A literature search was conducted in MEDLINE and EMBASE via the OVID platform using the key words “interstitial lung disease,” “ILD,” “interstitial pneumonitis,” “pneumonitis,” and “alveolar proteinosis.” In addition, key words for the two mTOR inhibitors everolimus and sirolimus were used (“mTOR,” “m-TOR,” “everolimus,” “Certican,” “RAD001,” “RAD 001,” “RAD-001,” “sirolimus,” “rapamycin,” “Rapamune,” “AY 22989,” “SILA 9268A,” “WY-090217,” and “proliferation signal inhibitor”). English language publications reporting information regarding diagnosis and/or clinical management of ILD in solid organ transplant recipients were included. Conference abstracts, review articles, commentaries, and publications that did not report information on diagnosis or clinical management of ILD were excluded. The literature search included references up to May 2014.

## 3. Results

### 3.1. Cases of ILD in Clinical Trials of Everolimus

**Figure 1:**
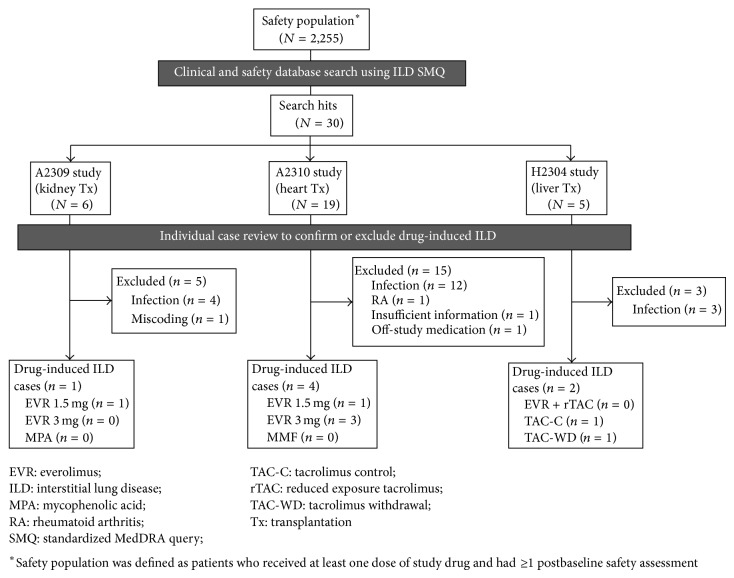
Clinical and safety database search flow diagram (studies A2309, A2310, and H2304).

Applying the SMQ search strategy to the entire study safety population (including control patients), a total of 30 events were retrieved from the clinical and safety databases (Figure 1). Following detailed medical review, drug-induced ILD was excluded in 23 events on the basis of the predefined exclusion criteria. Evidence of lung or systemic infection was found in 19 of these events. The other four cases were excluded because in one patient ILD was due to rheumatoid arthritis with lung involvement, one event was erroneously coded as ILD during database processing but was in fact a case of renal interstitial fibrosis, one case had insufficient information to permit accurate diagnosis, and one patient had stopped study medication 168 days prior to diagnosis of ILD. The remaining seven cases were determined to be drug-induced ILD. Six were confirmed as everolimus-induced ILD (four in heart transplant patients and one each in kidney and liver transplant patients) and one case was identified in a patient in the tacrolimus control arm of the liver transplant study.

### 3.2. Case Descriptions of Everolimus-Induced ILD

#### 3.2.1. Kidney Transplantation

*Case 1*. A 47-year-old man underwent living-related renal transplantation for end-stage renal disease due to hypertension and nephrosclerosis. He was randomized to receive everolimus 1.5 mg/d, with basiliximab induction, cyclosporine, and steroids. Eleven months later, he developed mild intermittent dyspnea. The everolimus trough level was 5.3 ng/mL. After two months, a bronchoscopic biopsy confirmed a diagnosis of pulmonary alveolar proteinosis. He continued to receive everolimus. Four months after the initial symptoms of dyspnea, the patient died of pneumonia and sepsis.

#### 3.2.2. Heart Transplantation

*Case 1*. A 65-year-old man underwent heart transplantation due to coronary artery disease. He was randomized to everolimus 3 mg/d, with basiliximab induction, cyclosporine, and steroids. After 15 months, the patient was hospitalized with fever and persistent cough. The latest available everolimus trough level (at month 12) was 11.9 ng/mL. Chest X-ray demonstrated subtle opacities in the right lower lobe and a CT scan suggested interstitial pneumonitis. A cardiac biopsy ruled out rejection. He was treated with clotrimazole, valganciclovir, and prednisone, with no response. Everolimus was discontinued and he was switched to MMF. Cyclosporine was continued. Five days later, a second CT scan showed marked interval clearing of previous interstitial densities at the lung bases. The patient was discharged after one week with his condition improving. The interstitial pneumonitis was considered ongoing at month 24 when the study ended.

*Case 2*. A 54-year-old man underwent heart transplantation due to coronary artery disease. He was randomized to everolimus 3 mg/d with basiliximab induction, cyclosporine, and steroids. Three months later, he was admitted with a four-week history of progressive dyspnea. His everolimus trough level was 8.9 ng/mL. Chest X-ray and CT scan were suggestive of severe interstitial pneumonitis. Everolimus was discontinued and he was switched to MMF and later to azathioprine. Cyclosporine and steroids were continued. The interstitial pneumonitis was considered to have resolved three weeks after everolimus discontinuation. 

*Case 3*. A 51-year-old man underwent heart transplantation due to idiopathic cardiomyopathy. He was randomized to everolimus 1.5 mg/d with rabbit antithymocyte globulin induction, cyclosporine, and steroids. Six months after transplantation, the patient developed dyspnea. Chest X-ray and CT scan showed no abnormalities. The everolimus trough level was 7.4 ng/mL. The dyspnea resolved after a few days but recurred three months later, requiring hospitalization. Cardiac biopsy ruled out rejection. An echocardiogram showed a left ventricular ejection fraction of 50–59%. The chest CT scan revealed bilateral ground glass opacities suggestive of interstitial pneumonitis. Everolimus was discontinued and the patient was switched to MMF. Cyclosporine and steroid were continued. No information on the outcome of the event was reported, but the patient was discharged from hospital two days after the diagnosis and was alive when the study ended. 

*Case 4*. A 61-year-old woman underwent heart transplantation due to coronary artery disease. She was randomized to everolimus 3 mg/d with basiliximab induction, cyclosporine, and steroids. Four weeks later, the investigator reported mild ILD, potentially related to everolimus. The everolimus trough level at that time was 10.4 ng/mL. However, no action was taken and the patient completed the study on everolimus. The event was considered ongoing at month 24 when the study ended.

#### 3.2.3. Liver Transplantation

*Case 1*. A 59-year-old man underwent liver transplantation due to hepatitis C. He was randomized to everolimus with tacrolimus withdrawal at month 4 and ongoing steroids. Seven months later, the patient was hospitalized with fever and suspicion of lung infection. Chest X-ray revealed pulmonary infiltrate in the left lung. The trough everolimus level was 11 ng/mL. His condition did not improve with broad-spectrum antibiotic treatment. Bronchoscopy with bronchoalveolar lavage showed nonspecific chronic inflammation of the bronchial mucosa. No positive cultures were obtained. Lung biopsy suggested ILD. Everolimus was discontinued and the patient was switched to MMF. He was treated empirically with amoxicillin-clavulanate and fluconazole. Four weeks later his respiratory condition worsened, requiring endotracheal intubation and drainage of left pleural effusion. Seven weeks after the first admission with respiratory symptoms, the patient died due to respiratory failure and refractory shock.

## 4. Literature Review

**Figure 2:**
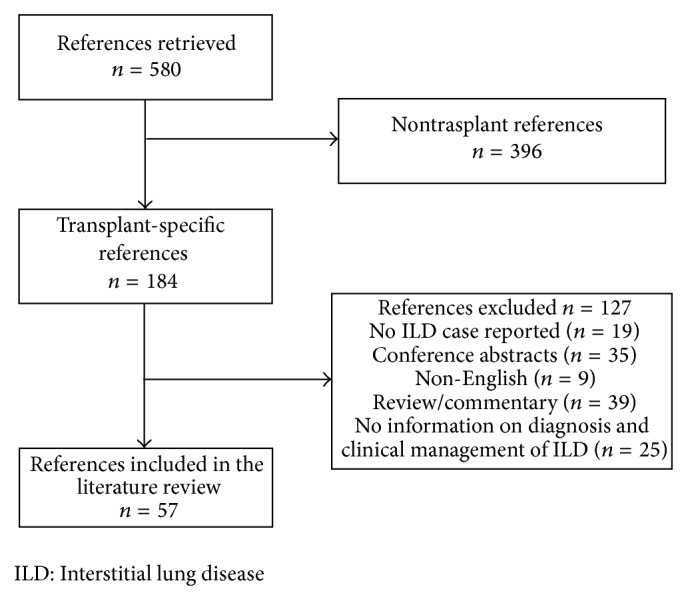
Literature search flow diagram.

In total, 57 publications were assessed as relevant and were included in the literature review (Figure 2). Of these, 45 publications provided detailed information on 68 cases of ILD (41 kidney, 17 heart, and 10 liver transplant recipients), as summarized in Table 2. The remaining 12 publications comprised 11 which reported 95 cases of ILD but supplied only limited information on individual events (Table 3) and one letter to the editor reporting a high level of information on 34 cases.

In the 68 cases for which detailed information was provided, 13 occurred in patients receiving everolimus (six kidney, six heart, and one liver transplant recipients) and 55 in patients receiving sirolimus (35 kidney, 11 heart, and nine liver transplant recipients). Both sirolimus and everolimus were generally administered in combination with other immunosuppressive agents (calcineurin inhibitors, MMF, and azathioprine), with or without steroids. Median age was 58 years (range 9 months–79 years) and the majority of patients were males (41/68, 60%). The terms used to describe the ILD event included pneumonitis, interstitial pneumonitis, organizing pneumonia, lymphocytic pneumonitis, lymphocytic alveolitis, pulmonary alveolar proteinosis, infiltrative pneumonia, alveolitis obliterans, allergic pneumonitis, interstitial granulomatous pneumonitis, and bronchiolitis obliterans. There was a wide variation in the time to ILD diagnosis after mTOR inhibitor initiation, ranging from as early as 5 days to six years (median four months). The most common presenting symptom was dyspnea. Other common presenting symptoms were cough and fever. Diagnosis was usually made based on chest X-ray and high-resolution CT scan. Biopsy, most commonly transbronchial, was used to diagnose ILD in around half of the cases.

Empirical antibiotics were administered in approximately 57% of the patients, with use of antifungal and antiviral treatment reported in a few patients. In most cases, testing of bronchoalveolar lavage for infectious agents was negative. In 31 of the 68 cases, steroids were administered to treat ILD. Discontinuation of mTOR inhibitor therapy led to resolution or improvement in 58 cases (85%). Of note, in one heart transplant recipient, ILD improved after everolimus discontinuation but recurred when everolimus was reinstituted [46]. In two heart transplant patients receiving sirolimus, the ILD was fatal [40, 42]. In both cases, respiratory distress developed and progressed rapidly after the start of sirolimus therapy. In seven cases, sirolimus was replaced by everolimus, which resulted in clinical improvement in all but one patient who had lymphocytic alveolitis and developed relapsing allergic pneumonitis after switching to everolimus [26]. In one kidney transplant recipient, ILD resolved after treatment with methylprednisolone despite continuing everolimus [31]. The letter to the editor by Singer et al. summarized 34 cases (32 kidney, one heart, and one liver transplant recipient) of interstitial pneumonitis associated with sirolimus [64]. These 34 instances also included the three cases reported by Morelon et al. [59], summarized in Table 3. In eight of the 34 cases, pneumonitis improved after sirolimus was discontinued. A fatal outcome was reported for four patients; the outcome in the remaining cases is not stated. Table 3 summarizes 11 publications that reported limited information on ILD in 95 mTOR inhibitor-treated patients (86 kidney and nine liver transplant recipients). As in the detailed case reports, ILD improved or resolved after mTOR inhibitor discontinuation or dose reduction.

## 5. Discussion

The diagnosis of drug-induced ILD remains challenging. The condition frequently remains unrecognized until the point at which pulmonary damage has become irreversible. Infections are the most frequent confounding factor, mimicking the clinical presentation of ILD and potentially superimposing changes on drug-induced damage to the lungs. A full understanding of drug-induced ILD is also hampered by a lack of standardized terminology, as demonstrated by the variety of terms used to describe the event in the published reports identified in our literature search.

mTOR inhibitors are a well-recognized cause of ILD. More cases of ILD have been reported with sirolimus than with everolimus in the literature, possibly due to earlier introduction and wider use of sirolimus, especially in kidney transplant recipients. Although ILD is considered to be a class effect of mTOR inhibitors, cases have been described in which symptoms improved or resolved when the patient was switched from sirolimus to everolimus [12, 26, 48]. This difference in toxicity between the two mTOR inhibitors has been linked to the more hydrophilic nature of everolimus [12, 26, 48]. Although the underlying mechanism leading to ILD in patients receiving mTOR inhibitors is not fully clarified, dose-dependent toxicity, T cell-mediated delayed-type hypersensitivity reaction, and idiosyncratic cell-mediated autoimmune response have been suggested as potential mechanisms [10, 21, 25, 40, 51, 60]. Morelon et al. suggested that both immune-mediated and direct toxicity may contribute to the development of ILD [60]. Dose-dependency of the effect remains controversial, since ILD has been reported in patients exposed to high, low, or within-target trough levels of everolimus and sirolimus [10, 16, 22, 25, 32, 42, 44, 45, 47, 53]. Evidence in favor of dose dependency comes from the oncology setting, where higher doses are used. In the pivotal Phase III clinical trials of everolimus (at a dose of 10 mg/day) in patients with advanced renal cell carcinoma, pancreatic neuroendocrine tumors, and hormone receptor positive breast cancer, pneumonitis was reported in 14%, 17%, and 12% of patients, respectively [65–67].

In the setting of solid organ transplantation, single-center studies have reported sirolimus-induced ILD in up to 16.7% of patients [57, 58, 61]. The incidence appears to be higher with late switch to sirolimus than with* de novo* use [22, 57, 61]. In addition to late switch, higher age, male gender, hypervolemia, allograft dysfunction, loading dose, high dose and high trough levels of sirolimus, and a prior increase in sirolimus dose or trough levels have been proposed as risk factors for the development of sirolimus-induced ILD [22, 40, 42]. For everolimus, single-center studies have reported ILD in up to 4.3% of patients [5, 6]. A recently published multicenter, retrospective, and case-cohort substudy of a randomized trial in renal transplant, recipients reported everolimus-induced ILD in 12.7% of patients (13/102). High incidence of ILD in this study could be partly explained by the combination of higher trough levels of everolimus (9.2, 10.8, and 14.5 ng/mL at 1, 6, and 12 months, resp.) than the recommended levels (3–8 ng/mL) and a higher incidence of underlying pulmonary disease at baseline in patients who developed ILD compared to those who did not. Importantly, everolimus discontinuation led to recovery from ILD in all the cases [55].

Our analysis of data from three large controlled clinical trials of everolimus in solid organ transplantation found the incidence of everolimus-related ILD to be 0.4% (six cases out of 1,473 patients). Of the six everolimus-related ILD cases, four occurred in heart transplant recipients with one each in kidney and liver transplant recipients. Of note, no cases of drug-related ILD were found in the cyclosporine/MPA control arms in the kidney and heart transplant studies, whereas one case of drug-related ILD was observed in the tacrolimus control arm in the liver transplant study (H2304). Across the three trials, the time to onset of symptoms was highly variable, ranging from four weeks to 15 months after the start of everolimus therapy, similar to the cases identified from the literature review. Dyspnea was the most common presenting symptom, consistent with the majority of published reports. Biopsy is considered the gold standard for diagnosis of ILD but, with the development of new imaging techniques, biopsy appears to have been reserved for cases in which diagnosis was uncertain after noninvasive techniques had been exhausted. In the three everolimus trials, CT scan was the most common diagnostic method, although a diagnostic biopsy was carried out in two of the six cases. Everolimus trough levels were within the protocol specified range in all the cases. Notably, four of the six ILD cases were reported in patients in the higher everolimus dose arm with an exposure to everolimus above the currently approved 3–8 ng/mL. Among the six cases identified in our analysis, ILD was fatal in two patients, confirming its life-threatening nature. The deaths occurred in one kidney transplant recipient in whom everolimus was not discontinued after diagnosis of ILD and one liver transplant recipient in whom a superimposed infection complicated the outcome despite everolimus discontinuation. Our analysis confirms the importance of prompt discontinuation of mTOR inhibitor therapy as soon as ILD is diagnosed. In three of four ILD cases in which everolimus was discontinued, ILD resolved or improved. This is further supported by the results of the literature search, which showed resolution or improvement in the majority of the ILD cases after mTOR inhibitor discontinuation.

The key strengths of our analysis are (1) a large safety database of prospectively collected information from randomized registration trials with uniform data requirements, (2) the search by SMQ terms for ILD, which is sufficiently wide to identify all potential cases of ILD while excluding terms referring to infectious lung events such as “infectious pneumonia” and “pulmonary infections,” and (3) the systematic case-by-case evaluation with prospectively defined criteria for exclusion of events other than drug-induced ILD. This approach may explain the lower incidence of ILD cases identified in these trials compared to the incidence reported by others. We acknowledge the limitations of our evaluation, including the fact that ILD detection was not a predefined objective of the studies, the potential bias of adverse event reporting by investigators in these open-label studies, and the point that medical assessment of the ILD cases was entirely dependent on the extent of information reported by the study investigators which, in some cases, was incomplete.

In conclusion, our research reinforces the complex and potentially fatal nature of mTOR inhibitor-induced ILD and highlights the need to include drug-induced ILD in the differential diagnosis of pulmonary conditions in transplant recipients receiving mTOR inhibitor therapy. A thorough evaluation by trained physicians to ascertain the diagnosis of drug-induced toxicity is recommended. Given the availability of immunosuppressive treatment options and the potential reversibility of the event, early diagnosis and rapid intervention to reduce or discontinue mTOR inhibitor therapy are key to the management of drug-induced ILD.

## Figures and Tables

**Table 1:** Standardized MedDRA query terms for identifying potential cases of ILD from clinical and safety databases.

Search terms	
Acute interstitial pneumonitis	Necrotizing bronchiolitis
Allergic granulomatous angiitis	Obliterative bronchiolitis
Alveolar proteinosis	Organizing pneumonia
Alveolar hemorrhage	Pneumonitis
Alveolitis	Progressive massive fibrosis
Allergic alveolitis	Pulmonary fibrosis
Acute eosinophilic pneumonia	Pulmonary necrosis
Chronic eosinophilic pneumonia	Pulmonary radiation injury
Diffuse alveolar damage	Pulmonary toxicity
Eosinophilia myalgia syndrome	Pulmonary vasculitis
Eosinophilic pneumonia	Radiation alveolitis
Fibrosing alveolitis	Radiation fibrosis—lung
Interstitial lung disease	Radiation pneumonitis
Lung infiltration	Transfusion-related acute lung injury
Necrosis of bronchioles	

**Table 2:** Summary of case reports identified in literature search.

	Age/sex	Terminology	Time after mTORi initiation	Presenting symptoms	Diagnosis	IS treatment	Action mTORi	Outcome
Kidney transplantation								
Amigues et al. 2005 [10]	48/M	Infiltrative pneumonia	1 month	Dyspnea, cough, and fever	CXR and HRCT	SRL + TAC + P	Disc.	Resolved
Bankar et al. 2006 [11]	56/M	Interstitial pneumonitis	2 months	Dyspnea and cough	CXR and HRCT	SRL + MMF	Disc.	Resolved
Calle et al. 2009 [12]	64/F	Interstitial pneumonitis	4.5 years	Dyspnea	CXR, Chest CAT	SRL + MPA + steroids	Switch to EVR	Improved
Chen et al. 2003 [13]	43/F	Interstitial pneumonia	Within 1 month	Cough and hemoptysis	CXR and HRCT	SRL (adjuvant) + CsA + MMF + MP	Disc.	Improved
Chhajed et al. 2006 [14]	65/F	Pneumonitis	3 months	Dyspnea and cough	CXR, CT, and TBB	SRL + TAC + P	Disc.	Improved
	48/F	Pneumonitis	8 months	Dyspnea	CXR, CT, and TBB	SRL + TAC + AZA + P	Disc.	Resolved
	61/M	Organizing pneumonia	2 years	Dyspnea, cough, fever, and hemoptysis	CXR, CT, and TBB	SRL + AZA	Disc.	Resolved
Filippone et al. 2011 [15]	61/M	Pneumonitis	6 years	Dyspnea and pleuritic chest pain	CT, bronchoscopy, and open lung biopsy	SRL + MMF + P	Disc.	Improved
Haydar et al. 2004 [16]	49/M	BOOP	18 months	Dyspnea and cough	CXR, CT, and bronchoscopy	SRL + CsA + P	Disc.	Resolved
	54/M	Pneumonitis	16 months	Dyspnea	HRCT and TBB	SRL	Disc.	Resolved
	63/M	Pneumonitis	7 months	Dyspnea, cough, and fever	CXR and HRCT	SRL + AZA + P	Disc.	Resolved
Huang et al. 2013 [17]	65/F	Pneumonitis	17 months	Dyspnea, cough, and fever	CXR, CT, and bronchoscopy	SRL + MMF + TAC + P	Disc.	Improved
	64/F	Pneumonitis	2 years	Dyspnea and cough	CXR and CT	SRL + MMF + TAC + P	Disc.	Improved
Kadikoy et al. 2010 [18]	49/F	PAP	3 years	Dyspnea and cough	CXR, CT, and bronchoscopy	SRL + MPA + P	Disc.	Improved
Kanaan et al. 2008 [19]	64/M	Interstitial pneumonitis	2 weeks	Dyspnea, cough, and fever	CXR and HRCT	SRL	Disc.	Resolved
Kirby et al. 2012 [20]	31/M	Organizing pneumonia	—	Dyspnea and cough	CT and TBB	SRL + MPA + CsA + P	Disc.	Improved
Mingos and Kane 2005 [21]	30/F	Interstitial pneumonitis	6 months	Dyspnea, cough, fever, and chest pain	Noncontrast CT and TBB	SRL + P	Disc.	Resolved
Morath et al. 2007 [22]	61/M	Interstitial pneumonitis	6 weeks	Dyspnea and fever	CXR, CT, and TBB	SRL + MP	Disc.	Improved
	67/M	Lymphocytic alveolitis	2 weeks	Dyspnea and cough	CXR, HRCT, and TBB	SRL + MMF + MP	Disc.	Resolved
	62/M	Interstitial pneumonitis	2 weeks	Dyspnea, cough, and fever	CXR and CT	SRL + MP	Disc.	Improved
	42/F	Interstitial pneumonitis	2 years	Dyspnea	CXR and CT	SRL + TAC + MP	Disc.	Improved
Nayak et al. 2004 [23]	54/M	Pneumonitis	2 months	Dyspnea	CXR and CT	SRL + MMF + P	Disc.	Resolved
Pedroso et al. 2007 [24]	34/F	PAP	2 years	Dyspnea, cough, fever, chest and pain	CXR, CT, and bronchoscopy	SRL + MMF + steroids	Disc.	Resolved
Pham et al. 2004 [25]	57/F	Pulmonary toxicity	2.5 months	Dyspnea, chills, and night sweats	CXR, HRCT, and wedge biopsy	SRL + low-dose TAC + P	Disc.	Improved
	42/F	Pulmonary toxicity	2.5 months	Dyspnea and fever	CXR, HRCT, and TBB	SRL + low dose CsA + P	Disc.	Improved
	55/F	Pulmonary toxicity	4 months	Dyspnea, cough, and hemoptysis	CXR, HRCT, and TBB	SRL + TAC	Disc.	Improved
Rehm et al. 2006 [26]	61/M	Pneumonitis	—	Dyspnea, fever, and pulmonary infiltrates	CXR, CT, and TBB	SRL + AZA + P	Switch to EVR	Improved
	55/F	Alveolitis obliterans	—	Dyspnea and cough	Bronchoscopy	SRL + MMF	Switch to CsA + MPA followed by EVR + MPA	Improved
	63/M	Allergic pneumonitis	—	Pulmonary symptoms	CXR and bronchoscopy	SRL + MMF	Switch to EVR	Improved
	51/F	Allergic interstitial pneumonitis	—	Cough and fever	TBB	SRL + MMF	Switch to EVR	Improved
	65/M	Lymphocytic alveolitis	—	—	TBB	SRL + MMF	Switch to EVR^††^	Improved
Sajjad et al. 2006 [27]	79/M	Interstitial lung disease	—	Dyspnea, cough, and chills	CXR, CT, HRCT, and TBB	SRL + P	Disc.	Improved
Shefet et al. 2004 [28]	51/F	Interstitial pneumonitis	11 months	Bilateral infiltrates chest X-ray	TBB	SRL + TAC + P	Disc.	Improved
Singh et al. 2009 [29]	15/F	Pneumonitis	4 years	Dyspnea	CT, bronchoscopy, and pulmonary biopsy	SRL + MMF	Disc.	Resolved
Ussavarungsi et al. 2012 [30]	53/F	Granulomatous pneumonitis	2 months	Dyspnea, fever, and hypoxia	CXR, CT, and thoracoscopic biopsy	SRL + CsA	Disc.	Improved
Carreño and Gadea 2007 [31]	56/F	BOOP	2 months	Dyspnea, cough, and fever	CXR and bronchoscopy	EVR	No action	Resolved
Alexandru et al. 2008 [32]	57/M	Pharmacological pneumonitis	3 months	Dyspnea, cough, and fever	CXR, CT, and lung biopsy	EVR + MMF	Disc.	Improved
Bouvier et al. 2009 [33]	74/M	Hypersensitivity pneumonitis	1 year	Dyspnea, cough, and fever	CXR, CT, and TBB	EVR	Disc.	Improved
Kurnatowska et al. 2010 [34]	59/F	Interstitial pneumonitis	6 weeks	Dyspnea and cough	HRCT and TBB	EVR + MMF + steroids	Disc.	Resolved
González et al. 2010 [35]	70/M	Interstitial pneumonitis	4 months	Dyspnea, cough, and fever	CXR, CT, and bronchoscopy	EVR + MPA + steroids	Disc.	Improved
Sułkowska et al. 2012 [36]	30/M	Pneumonitis	5 days	Dyspnea, fever, sore throat, desaturation, and respiratory insufficiency	CXR, HRCT, and TBB	EVR + MPA + P	Disc.	Resolved

Heart transplantation								
Chau and Chow 2006 [37]	47/M	Pneumonitis	3 months	Dyspnea, cough, and fever	CXR and HRCT	SRL + CsA + P	Disc.	Resolved
Delgado et al. 2006 [38]	58/M	Interstitial pneumonitis	3 years	Dyspnea and cough	CXR and HRCT	SRL + CsA + MMF + P	Disc.	Improved
	66/M	Interstitial pneumonitis	2 months	Cough and respiratory insufficiency	CXR and HRCT	SRL + MMF + P	Disc.	Resolved
	65/M	Interstitial pneumonitis	1.5 year	Cough and fever	HRCT	SRL + MMF + P	Disc.	Resolved
García-Luque et al. 2008 [39]	70/M	Interstitial pneumonitis	2 months	Dyspnea, cough, fever, and chest pain	CXR and HRCT	SRL + MMF	Disc.	Improved
	62/F	Interstitial pneumonitis	10 months	Dyspnea, cough, and fever	CXR and HRCT	SRL + MMF	Disc.	Improved
	55/M	Interstitial pneumonitis	4 months	Fever and cough	CXR, HRCT	SRL + MMF	Disc.	Improved
Garrean et al. 2005 [40]	64/F	Pulmonary toxicity	2 weeks	Dyspnea, fever, and hemoptysis	CXR, CT, and bronchoscopy	SRL + MPA + P	—	Death
Hamour et al. 2006 [41]	59/M	Pneumonitis	Progressively during 2 months	Dyspnea and fever	CXR, HRCT	SRL + CsA	Disc.	Resolved
Manito et al. 2004 [42]	52/M	Interstitial pneumonitis	10 days	Respiratory distress	CXR	SRL + MMF + P	Dose red. and disc.	Death
McWilliams et al. 2003^*^ [43]	32/M	Organizing pneumonia/pneumonitis	1 month	Dyspnea and lung function restriction	CXR, HRCT, and TBB	SRL + P	Disc.	Improved
David et al. 2007 [44]	71/M	Bronchiolitis obliterans	—	Fever and respiratory symptoms	HRCT	EVR + MMF + P	Disc.	Improved
Exp$\overset{´}{\text{o}}$sito et al. 2008 [45]	45/M	Organizing pneumonia	4 months	Dyspnea, cough, fever, and pleuritic chest pain	CXR, CT, and TBB	EVR + AZA + P	Disc.	Improved
	66/M	Organizing pneumonia	2 months	Cough, fever, and pleuritic chest pain	CXR and TBB	EVR + AZA	Disc.	Improved
Otton et al. 2009 [46]	32/M	Pneumonitis	2 weeks	Dyspnea	CXR, CT, and bronchoscopy	EVR + CsA + MMF + P	Disc.	Improved
	68/M	Pneumonitis	3 months	Dyspnea and hemoptysis	CXR, HRCT, and bronchoscopy	EVR + CsA + MMF + P	Disc.	Improved. Second episode after EVR reinstitution
	60/M	Pneumonitis	6 months	Dyspnea	HRCT	EVR + AZA + CsA + P	Disc.	Improved

Liver transplantation								
Avitzur et al. 2003 [47]	8/F	Interstitial granulomatous pneumonitis + PTLD	9 months	Dyspnea	CXR, CT, and open lung biopsy	SRL with CsA tapering and discontinuation	Disc.	Improved
De Simone et al. 2007 [48]	62/F	Pneumonitis	9 months	Dyspnea and fever	CXR	SRL monotherapy	Switch to EVR	Resolved
Feagans et al. 2009 [49]	55/M	Pulmonary toxicity	8 months	Dyspnea, cough, and fever	CXR, CT, and lung biopsy	SRL + CsA + steroids	Disc.	Resolved
	60/M	Pulmonary toxicity	37 months	Dyspnea, cough, and fever	CXR and CT	SRL + MPA + P	Disc.	Improved
Gupte et al. 2007^†^ [50]	2/F	Pulmonary toxicity	5 months	Dyspnea and cough	CXR and HRCT	SRL + TAC + P	Disc.	Improved
	9 month/M	Pulmonary toxicity	7 months	Dyspnea and cough	CXR and HRCT	SRL + TAC + P	Disc.	Improved
Howard et al. 2006 [51]	73/F	Pulmonary hypersensitivity	2 weeks	Dyspnea and cough	CXR, CT, and TBB	SRL + AZA + P	Disc.	Resolved
Lennon et al. 2001 [52]	49/M	Pneumonitis	5 months	Dyspnea and cough	CXR and CT	SRL + AZA + steroids	Disc.	Resolved
P$\overset{´}{\text{e}}$rez et al. 2007 [53]	62/M	Interstitial pneumonitis	3 months	Dyspnea, cough, and fever	CXR and CT	SRL + MMF + steroids	Disc.	Resolved
Schrader et al. 2010 [54]	63/F	Bronchiolitis obliterans	2 months	Dyspnea	CT, bronchoscopy, and peripheral lung biopsy	EVR + stepwise reduction of CsA + MMF + steroids	Disc.	Resolved

^*^Heart-lung transplantation; ^†^Combined small bowel and liver transplantation; ^††^EVR discontinued due to relapsing allergic pneumonitis.

AZA, azathioprine; BOOP, bronchiolitis obliterans organizing pneumonia; CAT, computed axial tomography; CsA, cyclosporine; CT, computed tomography; CXR, chest X-ray; Disc., discontinued; EVR, everolimus; HRCT, high resolution computed tomography; MMF, mycophenolate mofetil; MPA, mycophenolate; MP, methylprednisolone; mTORi, mammalian target of rapamycin inhibitor; P, prednisolone; PAP, pulmonary alveolar proteinosis; PTLD, posttransplant lymphoproliferative disorder; SRL, sirolimus; TAC, tacrolimus; TBB, transbronchial biopsy.

**Table 3:** Summary of publications with limited case-by-case information.

Reference	Patients with ILD	mTORi	Action mTORi	Outcome
Kidney transplantation				
Baas et al. 2014 [55]	13	EVR	Disc.	Recovered
Bertolini et al. 2011 [56]	1	EVR	Not stated	Not stated
Champion et al. 2006 [57]	24	SRL	Disc.	Recovered
Errasti et al. 2010 [5]	8	SRL (*n* = 3) and EVR (*n* = 5)	Disc.	Recovered
Lee et al. 2012 [58]	12	SRL	Dose red. (4) and disc. (8)	Resolved
Morelon et al. 2000 [59]	3	SRL	Disc.	Resolved
Morelon et al. 2001 [60]	8^*^	SRL	Dose red. (1) and disc. (7)	Resolved
Rodríguez-Moreno et al. 2009 [6]	6	SRL (*n* = 1) and EVR (*n* = 5)	Disc.	Recovered
Weiner et al. 2007 [61]	11	SRL	Dose red. (6) and disc. (5)	Resolved
Liver transplantation				
Morcos et al. 2012 [62]	5	SRL	Disc.	Resolved
Roberts et al. 2007 [63]	4	SRL	Disc.	Resolved

^*^Includes 3 cases described in Morelon 2000 [59] publication.

EVR, everolimus; Disc., discontinued; ILD, interstitial lung disease; mTORi, mammalian target of rapamycin inhibitor; SRL, sirolimus.
